# Supplementary material for: Real-time visualisation of the intracellular dynamics of conjugative plasmid transfer
Source: Nat Commun. 2023 Jan 18;14:294. doi: 10.1038/s41467-023-35978-3 (PMC9849209; doi:10.1038/s41467-023-35978-3)
Supplement: Supplementary file 3 — Description of Additional Supplementary Files [file 41467_2023_35978_MOESM3_ESM.pdf]

## Description of Additional Supplementary Files:

**Movie S1:** Microfluidic time-lapse imaging of conjugation showing the production of leading proteins sfGFP fusion as respect to the formation of a mCh-ParB focus in transconjugant cells. Donors carry the F derivative with the indicated sfGFP fusion, and recipient cells produce mCh-ParB from the pSN70 plasmid. Before plasmid transfer, the mCh-ParB is diffuse in the recipient cells and mCh-ParB focus formation reports the ssDNA-todsDNA conversion after ssDNA acquisition. Merge of phase contrast, mCherry and sfGFP channels are shown. Cells were grown in M9-CAA at 37°C, images were taken every 5 min. Scale bar 1 µm and time in minutes are indicated.

**Movie S2:** Microfluidic time-lapse imaging of conjugation showing the production of maintenance and Tra proteins sfGFP fusions as respect to the formation of a mCh-ParB focus in transconjugant cells. Donors carry the F derivative with the indicated sfGFP fusion, and recipient cells produce mCh-ParB from the pSN70 plasmid. Before plasmid transfer, the mCh-ParB is diffuse in the recipient cells and mCh-ParB focus formation reports the ssDNA-to-dsDNA conversion after ssDNA acquisition. Merge of phase contrast, mCherry and sfGFP channels are shown. Cells were grown in M9- CAA at 37°C, images were taken every 5 min. Scale bar 1 µm and time in minutes are indicated.

**Movie S3:** Microfluidic time-lapse imaging of conjugation showing the production SsbF-sfGFP fusion as respect to the formation of a mCh-ParB focus in transconjugant cells. Donors carry the F ssbF-sfgfp but do not exhibit green fluorescence as SsbF-sfGFP is not produced. SsbFsfGFP is initially diffuse in the recipient cells when the mCh-ParB focus is already present. SsbF-sfGFP forms foci concomitantly with mCh-ParB focus duplication events. From left to right, merge of phase contrast, mCherry and sfGFP channels; merge of mCherry and sfGFP channels; mCherry channel; and sfGFP channel are shown. Cells were grown in M9-CAA at 37°C, images were taken every 5 min. Scale bar 1 µm and time in minutes are indicated.
